# Clinical Practice Guidelines Using GRADE and AGREE II for the Impact of Genetic Variants on Plasma Lipid/Lipoprotein/Apolipoprotein Responsiveness to Omega-3 Fatty Acids

**DOI:** 10.3389/fnut.2021.768474

**Published:** 2022-02-14

**Authors:** Justine Keathley, Véronique Garneau, Valérie Marcil, David M. Mutch, Julie Robitaille, Iwona Rudkowska, Gabriela Sofian, Sophie Desroches, Marie-Claude Vohl

**Affiliations:** ^1^Centre Nutrition, Santé et Société (NUTRISS), Institut sur la Nutrition et les Aliments Fonctionnels (INAF), Université Laval, Québec City, QC, Canada; ^2^School of Nutrition, Université Laval, Québec City, QC, Canada; ^3^Research Centre, Sainte-Justine University Health Centre, Montréal, QC, Canada; ^4^Department of Nutrition, Université de Montréal, Montréal, QC, Canada; ^5^Department of Human Health and Nutritional Sciences, University of Guelph, Guelph, ON, Canada; ^6^Endocrinology and Nephrology Unit, Centre Hospitalier Universitaire de Québec-Université Laval Research Center, Québec City, QC, Canada; ^7^Department of Kinesiology, Université Laval, Québec City, QC, Canada; ^8^Library, Université Laval, Québec City, QC, Canada

**Keywords:** nutrigenetics, nutrigenomics, lipids, lipoproteins, apolipoproteins, omega-3, EPA, DHA

## Abstract

**Background:**

A recent systematic review, which used the GRADE methodology, concluded that there is strong evidence for two gene-diet associations related to omega-3 and plasma triglyceride (TG) responses. Systematic reviews can be used to inform the development of clinical practice guidelines (CPGs).

**Objective:**

To provide guidance for clinical practice related to genetic testing for evaluating responsiveness to dietary/supplemental omega-3s and their impact on plasma lipids/lipoproteins/apolipoproteins.

**Design:**

Using the results of the abovementioned systematic review, the first CPGs in nutrigenetics were developed using the established GRADE methodology and AGREE II approach.

**Results:**

Three clinical practice recommendations were developed. Most gene-diet associations identified in the literature lack adequate scientific and clinical validity to warrant consideration for implementing in a practice setting. However, two gene-diet associations with strong evidence (GRADE quality: moderate and high) can be considered for implementation into clinical practice in certain cases: male *APOE*-E4 carriers (rs429358, rs7412) and TG changes in response to the omega-3 fatty acids eicosapentaenoic acid (EPA) and/or docosahexaenoic acid (DHA) as well as a 31-SNP nutrigenetic risk score and TG changes in response to EPA+DHA among adults with overweight/obesity. Ethical and regulatory implications must be considered when providing *APOE* nutrigenetic tests given the well-established link between *APOE* genetic variation and Alzheimer's Disease.

**Conclusion:**

Most of the evidence in this area is not ready for implementation into clinical practice primarily due to low scientific validity (low quality of evidence). However, the first CPGs in nutrigenetics have been developed for two nutrigenetic associations with strong scientific validity, related to dietary/supplemental omega-3 and TG responses.

## Introduction

Genetic testing for personalized nutrition has been available for several years to the general public through direct-to-consumer services and healthcare professionals (HCPs). This can be referred to as nutrigenetics—the study of the influence of genetic variability and dietary/supplemental intake on subsequent health outcomes (1). There is considerable debate in the field about whether nutrigenetic testing is ready for “prime time” (2), but it has been suggested that there are certain gene-diet associations with strong evidence that could be considered for implementation into clinical practice (3, 4). However, systematic reviews that include evidence grading have yet to be comprehensively conducted in the field to inform on levels of evidence for specific nutrigenetic associations. Moreover, clinical practice guidelines (CPGs) in nutrigenetics do not yet exist. With this in mind, the development of CPGs by expert panels is urgently needed in order to guide best practice for clinicians and industry alike.

Recently, the first two systematic reviews with evidence grading were published in the field of nutrigenetics (5, 6). These reviews were specific to nutrigenetic contributions to cancer risk (6) and nutrigenetics, omega-3 intake and plasma lipids/lipoproteins/apolipoproteins (5). The ultimate goal of evidence grading is to determine if there is sufficient evidence (or not) to guide nutrition recommendations. While most of the existing evidence related to these two review topics was deemed low quality (or “weak”) (5, 6), there were three identified gene-diet associations with moderate- and high-quality evidence (5, 6). These include the 10p14 locus and processed meat consumption related to colorectal cancer risk, triglyceride (TG) responsiveness to eicosapentaenoic acid (EPA) and/or docosahexaenoic (DHA) based on *APOE* (rs429358, rs7412) genetic variants in men, and TG responsiveness to EPA+DHA based on a 31-single nucleotide polymorphism (SNP) nutrigenetic risk score (nutri-GRS) (5, 6). Therefore, it is possible that these specific nutrigenetic associations could be integrated into a clinical setting. However, scientific validity is just one component when considering the potential implementation of genetic testing into clinical practice (7, 8).

The development of CPGs requires considering multiple factors such as preferences of the target patient population, generalizability and consideration of other management options, and tools have been developed to help ensure that all relevant factors have been considered and thus, that guidelines are high-quality. For example, the Appraisal of Guidelines, Research and Evaluation (AGREE) instrument was established to help guide comprehensive CPG development. A more recent iteration entitled AGREE II has been published (9) and used in the field of nutrition (10–12).

The specific objective of this study is to provide guidance for clinical practice and/or recommendations for future research related to genetic variation, dietary/supplemental omega-3 and plasma lipids/lipoproteins/apolipoproteins using the established GRADE methodology and AGREE II approach (9, 13–15). Developing CPGs related to nutrigenetics of colorectal cancer risk was deemed to be outside the scope of these specific reviews given the different subject area, but this is an important topic for future CPG development. Specific lipid and lipoprotein outcomes of interest to the present CPGs were: high-density lipoprotein (HDL)-cholesterol, low-density lipoprotein (LDL)-cholesterol, LDL particle size, total cholesterol, apolipoproteins, and TG. Ultimately, this CPG will aim to support evidence-based practice for the personalized nutrition management of plasma lipids/lipoproteins/apolipoproteins in adults using omega-3s, based on individual genetic variation. We expect that these CPGs will lead to improved evidence-based practice in genetic testing for personalized nutrition related to gene-omega-3 associations modifying plasma lipids/lipoproteins/apolipoproteins. The intention is to provide this information to relevant stakeholders such as clinicians, industry and policy makers, to help them determine which nutrigenetic tests could be offered to patients, and in which circumstances/situations. The guidelines are applicable to male and female adults (18 years and older), excluding pregnancy and lactation.

## Methods

CPGs were developed by following the steps outlined in the GRADE Series' guideline on moving from evidence to recommendations (13–15) as well as the AGREE II for CPG development (9) after the systematic review process (registered with PROSPERO CRD42020185087).

All relevant components of AGREE II were included in the CPG development (Supplementary Table 1). The four key factors for determining the strength of recommendations, according to the GRADE approach, were also considered and included. These factors include: quality of the evidence, balancing desirable vs. undesirable consequences, values and preferences, as well as resource use (13–15). Four authors (JK, VG, SD, M-CV) developed the guidelines; three of these authors are registered dietitians (JK, SD, VG), one is a postdoctoral fellow (JK), and two are university professors specializing in either nutrition knowledge synthesis (SD) or nutrigenetics (M-CV). Two authors (JK and VG) were primarily responsible for the systematic review and evidence evaluation, with oversight and guidance from two authors (SD and M-CV) (5). One author (JK) took primary responsibility for drafting the CPGs. All other authors (DMM, JR, IR, VM, GS) revised and approved the final CPGs. The authors provided open-ended comments and suggestions, including those related to the facilitators and barriers to implementing recommendations, which were then reviewed by the CPG development committee (JK, VG, SD, M-CV). Any disagreement regarding the suggested revisions was reached through a discussion of key considerations, and then voting on a final decision among all guideline authors. There were three recommendations that were discussed and modified accordingly by the group, which included specifying EPA and/or DHA for the *APOE*-related recommendations; specifying EPA+DHA for the nutri-GRS related recommendations; and clarifying that ALA has not been shown to have beneficial effects on TG levels.

Adherence to these CPGs can be monitored/evaluated as needed by conducting research assessing the proportion of healthcare professionals (HCPs) and companies abiding by the recommendations presented herein for their nutrigenetic tests. As new evidence emerges, these CPGs should be updated, using the same methodology.

## Results

### Quality of Evidence

In general, the higher the quality of evidence, the stronger the clinical recommendation (15). The strengths and limitations of the body of evidence have been detailed in the previously conducted systematic review (5). The vast majority of genetic variant(s) identified in the systematic review (5) had weak evidence (GRADE evidence quality rating: low or very low) for their impact on plasma lipid/lipoprotein/apolipoprotein responsiveness to omega-3 fatty acids and thus guidelines for practice were not developed for these nutrigenetic associations. At this time, these should not be considered for incorporation into clinical practice given their low scientific validity. The systematic review and evidence GRADE process previously conducted (5), indicated strong evidence for TG responsiveness to EPA and/or DHA based on *APOE* (rs429358, rs7412) genetic variants in men (GRADE evidence quality rating: moderate) (16–24), and TG responsiveness to EPA+DHA based on a 31-SNP nutri-GRS in men and women with overweight/obesity (GRADE evidence quality rating: high) (25, 26). It should be noted that there is no evidence for the effectiveness for TG responsiveness to alpha-linolenic acid (ALA) for either of these two nutrigenetic associations. Furthermore, there is no evidence to support nutrigenetic associations related to ALA intake and other plasma lipids/lipoproteins/apolipoproteins (5). A more thorough description of the strengths and limitations of the body of evidence has been detailed previously (5).

### Balance Between Desirable and Undesirable Effects (Potential Benefits vs. Potential Harms)

Trade-offs between desirable and undesirable consequences of alternative TG management strategies (nutrigenetic intervention vs. other population-based strategies for TG management), as well as risks and benefits of omega-3 consumption and nutrigenetic testing were considered herein.

In addition to EPA+DHA consumption, effective lifestyle strategies for maintaining healthy TG levels can also include reducing the intake of refined sugar/carbohydrates, alcohol, *trans* fatty acids, and/or increasing physical activity (27). These strategies should be considered and recommended to patients on an individualized basis to maintain healthy TG levels, in addition to nutrigenetic-based advice for omega-3s if the HCP and patient decide to complete such genetic testing. Drug therapy may also be warranted in some patients, but this is beyond the scope of these nutrition CPGs. Given the large interindividual variability observed in plasma TG responsiveness to EPA+DHA (18, 28, 29), nutrigenetic testing can help evaluate which patients will benefit the most from EPA+DHA consumption for TG management. Moreover, providing a “one-size fits all” recommendation for all patients to consume EPA+DHA to reduce TG may have undesirable effects and risks for approximately one third of the population, who have been shown to be non-responders, or adverse responders (i.e., exhibit increases in TG) to EPA+DHA intake for TG changes (25, 26). In addition to an increase in TG in adverse responders, an insulin sensitivity lowering was observed following an n-3 supplementation in 23% of the subjects and a GRS built with 8 of the lead SNPs associated with HOMA-IR changes in this sample accurately predicted the occurrence of this adverse effect (30). The harms of a nutrigenetic intervention for omega-3 and TGs were deemed to be negligible, but genetic testing could be a way to prevent adverse effects of an EPA and/or DHA supplementation intervention, even for people taking n-3 for other conditions. Also, important ethical considerations for *APOE* genetic testing exist and must be considered. Compared to non-carriers, carriers of *APOE-*E4 have a 15 times greater risk of developing Alzheimer's disease (31). As such, ethical and regulatory considerations with *APOE* testing may present barriers to implementation. These implications are discussed in depth in CPG recommendation #1, below.

While there are some concerns about omega-3 supplementation potentially leading to mild adverse effects on certain other lipids and non-lipid biochemical markers (27, 32), as well as gastrointestinal discomfort and skin abnormalities in some patients, health organizations generally consider omega-3s to be safe and well-tolerated (32). In addition, cardiovascular guidelines recommend screening for dyslipidemia starting at age 40 or earlier if the patient has risk factors (e.g., high body mass index), so in many adults, plasma lipid, and other biochemical markers should be regularly monitored (33). Moreover, elevated plasma TG is associated with CVD risk and therefore TG reduction (which can be achieved through higher omega-3 intake in certain patients) has several potential health benefits (27, 34). Omega-3 intake has also demonstrated additional health benefits such as improving major depressive disorder in conjunction with other therapies (35–37), potentially improving cognitive function in very mild Alzheimer's disease (37, 38), and improving outcomes of inflammatory diseases (39, 40). To mitigate any potential risks, while consuming enough omega-3s to result in possible health benefits, the United States Food and Drug Administration (FDA) states that EPA+DHA supplementation should not exceed 5.0 g/day (41). However, it should also be noted that dosages of 3.0 g/day EPA+DHA are considered pharmacological dosages by the American Heart Association (42); this was taken into consideration in developing the CPG recommendations. In Canada, EPA+DHA omega-3 supplementation is not listed as a scheduled drug at any dosage level, but icosapent ethyl (a pure EPA) is considered a Schedule I drug according to the National Association of Pharmacy Regulatory Authorities and therefore limits its implementation in practice to HCPs who can provide prescriptions such as medical doctors (43). Therefore, location-specific regulatory requirements should be taken into consideration by the HCP recommending EPA+DHA to their patients. Regulations may differ depending on the type of HCP recommending EPA+DHA to the patient. Given the above considerations, it was determined by the authors of the present CPGs that overall the potential benefits outweighed the risks of taking these omega-3 fatty acids (EPA+DHA) up to this dosage. HCPs should still assess patients for any contraindications of omega-3 consumption such as fish allergy, adverse reactions, or drug-nutrient interactions (e.g., taking omega-3 supplements in combination with anticoagulant medications) (41). In addition, it is possible to achieve omega-3 (EPA+DHA) intake targets through food sources alone such as fish. For example, 150 g of Atlantic salmon contains just over 3 g of EPA+DHA (44), but some patients may still prefer omega-3 supplementation.

Overall, the balance between desirable and undesirable effects of a nutrigenetic intervention vs. standard population-based nutrition care regarding the use of omega-3 fatty acids (EPA+DHA) to reduce TG was deemed to be “important not critical” using the GRADE terminology (13).

### Values and Preferences

The information described in this section was used primarily to determine whether a weak/conditional (level 2) or strong (level 1) recommendation would be made in the present CPGs, as well as the potential caveats of these recommendation(s). Values and preferences of the target population (adult consumers and potential consumers of genetic testing for personalized nutrition) as well as HCPs were assessed through a literature review, which overall indicated that there is substantial interest globally in genetic testing for personalized nutrition (45–49). Consumers express interest in genetically-guided nutrition advice, especially if such advice provides advantageous recommendations that could be implemented into one's daily routine (48, 50); omega-3 supplementation provides an example of this type of advice. There have, however, been some concerns raised by consumers about genetic testing for personalized nutrition (45). Consumers have expressed their concern for the potential for companies to be more interested in financial gain rather than health (50). In addition, consumers have expressed concerns about nutrigenetic results being unclear or inaccurate, and preferred to undergo nutrigenetic testing through a HCP, such as a registered dietitian (45, 50). However, only a subset of registered dietitians (between 24 and 33% depending on their practice sector) perceives they have the knowledge to integrate nutrigenomics in their practice (51). Still, 50% of registered dietitians who recently received their licenses know about nutrigenetic testing compared to 12% of registered dietitians who have practiced for 25 years or more. This demonstrates an increasing interest in nutritional genomics among (certain) HCPs. Coupled with a similar interest among patient populations, these are considered facilitators to providing nutrigenetic-guided recommendations for omega-3s and TG responsiveness.

### Resource Use

The panel deemed it important to consider resource use for the present recommendations (in determining strength and direction), although this is considered optional in the GRADE approach (15). Resource use was considered through literature review and discussion among the guideline developers. There are financial costs associated with both omega-3 consumption (supplementation and through food sources) and nutrigenetic testing, which should be considered. The cost of nutrigenetic testing is highly variable and has been documented at between $90 and $450 CDN (52). Given this, evidence-based personalized nutrition advice should only be implemented if the patient is willing and able to cover their costs. Some HCPs are already offering genetic testing for personalized nutrition, and interest for genetic testing among consumers is high (53). However, it should be noted that several HCPs do not feel competent in the field of nutrigenetics and would thus require further training in the field prior to implementing this personalized nutrition approach in their practice (51, 54). In some cases, patients may have already completed a genetic test for the evidence-based SNPs and may bring their genotype results to a HCP for interpretation. If all SNPs have been tested, the 31-SNP nutri-GRS (detailed in Supplementary Table 2) could then be calculated and used by HCPs to interpret raw data from other genetic tests. A similar approach could be used for the two *APOE* SNPs. In other cases, a patient may be looking to complete genetic testing for these specific SNPs, and the feasibility of this will depend on what is offered by genetic testing laboratories and companies. With CPG development, the hope/goal is for companies to use the CPGs to develop evidence-based tests that can be offered to patients. Other additional resources beyond standard nutrigenetic care would include the initial investment of time required for the HCP to learn and understand the ethical and regulatory implications of *APOE* testing in their specific setting.

### Overall Recommendations Including Direction and Strength (Evidence to Recommendations Synthesis)

An “Evidence to recommendations framework” (15) is provided in Table 1. Recommendations 1 and 2 are conditional on the HCP offering the genetic testing being adequately trained in nutrigenetics (i.e., being competent in this area of nutrition care); this is considered a facilitator to implementation. These recommendations are further conditional on a patient's willingness to undergo genetic testing, including incurring any associated costs, which may be a barrier for patients. It is important to recognize that, similar to a personalized nutrition approach, the decision to undergo nutrigenetic testing should also be individualized and patient centered. The generalizability of the tests should also be noted (Table 2). Given these caveats, recommendations 1 and 2 were considered conditional (GRADE level 2), while recommendation 3 was considered “strong” (GRADE level 1). The following recommendations intend to allow for more targeted dietary advice specific to the individual in an effort to optimize cardiovascular health through TG management/prevention, in an evidence-based manner.

**Table 1:** Evidence to recommendations framework.

**Question/recommendation:** Should nutrigenetic testing for plasma lipid/lipoprotein/apolipoprotein* responsiveness to dietary/supplemental omega-3 be a component of clinical practice?
**Patient population:** Adult males and females (generalizability further detailed in Tables 1a–c, 2)
**Intervention:** Dietary/supplemental omega-3

**Table 1a:** *APOE* (rs429358 and rs7412) genetic testing to evaluate the TG responsiveness to dietary/supplemental EPA+DHA in males.

**Decision domain**	**Judgment**	**Reason for judgment**	**Subdomains influencing judgment**
Quality of evidence • Is there high or moderate quality evidence?	Yes ⊠ No □	⊕⊕⊕⊖ Strong (moderate-quality) evidence suggests that adult males (but not females) with the APOE-E3/E4 or E4/E4 genotype (rs429358, rs7412) experience significant reductions in TG in response to 0.7–3.7 g/day of EPA and/or DHA. Higher dosages may have greater TG lowering effects.	4 RCTs and 5 single arm trials have been conducted to date (16–24). Serious indirectness, including differences in age, omega-3 dosage and type (even when considering studies with male study samples separate from male + female study samples), as well as some differences in the results led to rating down the quality of evidence. However, evidence of a dose-response gradient and plausible mechanism of action strengthened the quality of evidence (5).
Balance of desirable and undesirable outcomes • Given the best estimate of typical values and preferences, are you confident that the benefits outweigh the harms and burden or vice versa?	Yes ⊠ No □	The desirable consequences are notable (such as more targeted TG management strategies for improved cardiovascular health) with minimal to no undesirable consequences.	With approximately one third of the population being categorized as a non-responder or adverse responder to omega-3 for TG lowering (25, 26), there is a risk associated with giving one-size-fits-all, population-based advice. In general, omega-3 is considered safe up to 3.0 g/day of EPA+DHA.
Values and preferences • Are you confident about the typical values and preferences and are they similar across the target population?	Yes □ No ⊠	If a patient wishes to undergo this nutrigenetic test and consents to genotyping, they should have the option to do so. The test offered to the patient should be evidence-based and ethically incorporated into practice (see quality of evidence and resource use sections).	In general, the public expresses an interest in genetic testing for personalized nutrition (45–49), especially if this testing leads to lifestyle recommendations (48, 50) and is offered by a registered dietitian or other HCP (45, 50). Moreover, there is substantial demand for genetic testing among consumers (patients) (53). However, the choice to undergo this type of genetic testing will vary from person to person and some individuals have expressed concerns (50). Given the variability that some patients would choose nutrigenetic testing over population-based advice while others would not, this variability resulted in rating down the strength of the recommendation.
Resource use • Are the resources worth the expected net benefit from following the recommendation?	Yes ⊠ No □	Resources are required for the implementation of nutrigenetic testing into practice, but several nutrigenetic testing companies exist and many HCPs are already offering this type of testing in their practice. There is often a cost for patients, and HCPs should be adequately trained, particularly with respect to the caveats associated with *APOE* genetic testing.	Nutrigenetic testing has been integrated into clinical practice for many years (53). The CPG recommendations presented herein would help to strengthen existing tests available on the market and evidence-based practice among HCPs (and thus patient outcomes).
Overall strength of recommendation	Weak (conditional)	The guideline panel conditionally recommends that nutrigenetic testing for TG responsiveness to EPA and/or DHA omega-3s can be based on genetic testing of *APOE* SNPs (rs429358, rs7412) in adult male patients (but not females).	
Evidence to recommendation synthesis	The quality of evidence, risk vs. benefit analysis, and resource implications suggest a strong recommendation however the variability among patients in choosing to undergo genetic testing for personalized nutrition resulted in rating down the strength of the recommendation to “weak” (conditional).		

**Table 1b:** Use of the Vallée Marcotte et al. 31-SNP nutri-GRS to evaluate the TG responsiveness to dietary/supplemental EPA+DHA in males and females with overweight/obesity.

**Decision domain**	**Judgment**	**Reason for judgment**	**Subdomains influencing judgment**
Quality of evidence • Is there high or moderate quality evidence?	Yes ⊠ No □	⊕⊕⊕⊕ Strong (high-quality) evidence suggests that in adults with overweight/obesity, a 31-SNP nutri-GRS can evaluate TG responsiveness to EPA+DHA supplementation. Individuals with lower scores demonstrate greater responsiveness to EPA+DHA for TG lowering.	1 RCT and 1 single arm trial have been conducted to date (25, 26). While there was some indirectness between studies, this was limited to the type of omega-3 and group stratification. One study used EPA+DHA supplementation while the other intervened with EPA or DHA supplementation (separate) (25, 26). Group stratification differed with one study stratifying participants as responders or non-responders, and the other stratifying as responders, non-responders or adverse responders (25, 26). However, balancing these limitations with evidence of a gradient for the nutri-GRS and TG responsiveness to omega-3 supplementation as well as some evidence of a mechanism of action resulted in rating the evidence back up (5).
Balance of desirable and undesirable outcomes • Given the best estimate of typical values and preferences, are you confident that the benefits outweigh the harms and burden or vice versa?	Yes ⊠ No □	The desirable consequences are notable (such as more targeted TG management strategies for improved cardiovascular health) with minimal to no undesirable consequences.	With approximately one third of the population being categorized as a non-responder or adverse responder to omega-3 for TG lowering (25, 26), there is a risk associated with giving one-size-fits-all, population-based advice. In general, omega-3 is considered safe up to 3.0 g/day of EPA+DHA.
Values and preferences • Are you confident about the typical values and preferences and are they similar across the target population?	Yes □ No ⊠	If a patient wishes to undergo this nutrigenetic test and consents to genotyping, they should have the option to do so. The test offered to the patient should be evidence-based and ethically incorporated into practice (see quality of evidence and resource use sections).	In general, the public expresses an interest in genetic testing for personalized nutrition (45–49), especially if this testing leads to lifestyle recommendations (48, 50) and is offered by a registered dietitian or other HCP (45, 50). Moreover, there is substantial demand for genetic testing among consumers (patients) (53). However, the choice to undergo this type of genetic testing will vary from person to person and some individuals have expressed concerns (50). Given the variability that some patients would choose nutrigenetic testing over population-based advice, others would not; this variability resulted in rating down the strength of the recommendation.
Resource use • Are the resources worth the expected net benefit from following the recommendation?	Yes ⊠ No □	Resources are required for the implementation of nutrigenetic testing into practice, but several nutrigenetic testing companies exist and many HCPs are already offering this type of testing in their practice.	Nutrigenetic testing has been integrated into clinical practice for many years (53). The CPG recommendations presented herein would help to strengthen existing tests available on the market and evidence-based practice among HCPs.
Overall strength of recommendation	Weak (conditional)	The guideline panel conditionally recommends that the Vallée Marcotte et al. nutri-GRS (25, 26) can be used to evaluate TG responsiveness to 3.0 g/day of EPA+DHA in adults with overweight/obesity.	
Evidence to recommendation synthesis	The quality of evidence, risk vs. benefit analysis, and resource implications suggest a strong recommendation however the variability among patients in choosing to undergo genetic testing for personalized nutrition resulted in rating down the strength of the recommendation to “weak” (conditional).		

**Table 1c:** Genetic testing of variants for evaluating plasma lipid/lipoprotein/apolipoprotein* responses to dietary/supplemental omega-3 other than omega-3/TG responsiveness and *APOE* (rs429358 and rs7412) or the Vallée Marcotte et al. 31-SNP nutri-GRS.

**Decision domain**	**Judgment**	**Reason for judgment**	**Subdomains influencing judgment**
Quality of evidence • Is there high or moderate quality evidence?	Yes □ No ⊠	The evidence is generally weak (low or very low quality) for SNPs influencing plasma lipid/lipoprotein/apolipoprotein* responses to dietary/supplemental omega-3 other than *APOE* (rs429358 and rs7412) and the Vallée Marcotte et al. 31-SNP nutri-GRS further detailed in Tables 1a,b. There is moderate quality evidence to demonstrate a *lack of effect* for *APOE* (rs rs429358 and rs7412), omega-3 and total cholesterol in females (16–24, 55) as well as *PPARg2* (rs1801282), omega-3 and LDL cholesterol (56–60).	While several observational and interventional studies have demonstrated evidence of various SNPs influencing plasma lipid/lipoprotein/apolipoprotein* responses to dietary/supplemental omega-3, most have not yet been replicated (5). In those that have been replicated, reasons for downgrading the evidence include risk of bias, inconsistency, indirectness, and imprecision. However, many nutrigenetic associations had evidence of a mechanism of action (5).
Balance of desirable and undesirable outcomes • Given the best estimate of typical values and preferences, are you confident that the benefits outweigh the harms and burden or vice versa?	Yes □ No ⊠	Given the lack of scientific evidence, there are minimal to no desirable consequences. It is undesirable to provide nutrition advice that is not evidence-based.	A lack of scientific evidence would lead to the public receiving dubious nutrition advice, that is unlikely to lead to health benefits above and beyond population-based advice.
Values and preferences • Are you confident about the typical values and preferences and are they similar across the target population?	Yes ⊠ No □	If a patient wishes to undergo this nutrigenetic test and consents to genotyping, they should have the option to do so. However, the test offered to the patient should be evidence-based and ethically incorporated into practice (see quality of evidence and resource use sections).	In general, the public expresses an interest in genetic testing for personalized nutrition (45–49), especially if this testing leads to lifestyle recommendations (48, 50) and is offered by a registered dietitian or other HCP (45, 50). Moreover, there is substantial demand for genetic testing among consumers (patients) (53). However, the choice to undergo this type of genetic testing will vary from person to person and some individuals have expressed concerns (50). Despite consumer interest in nutrigenetics, the CPG panel is confident that patients would not wish to receive dubious (scientifically invalid) nutrition information and advice.
Resource use • Are the resources worth the expected net benefit from following the recommendation?	Yes ⊠ No □	Resources are required for the implementation of nutrigenetic testing into practice, but several nutrigenetic testing companies exist and many HCPs are already offering this type of testing in their practice.	Nutrigenetic testing has been integrated into clinical practice for many years (53). The CPG recommendations presented herein would help to strengthen existing tests available on the market and evidence-based practice among healthcare professionals.
Overall strength of recommendation	Strong	The panel strongly recommends *not* to provide personalized omega-3 recommendations for lipids, lipoproteins and apolipoproteins* based on genetic variation of any SNPs beyond the nutri-GRS developed by Vallée Marcotte et al. (25, 26), or *APOE* (rs429358 and rs7412) genotype = based on the evidence currently available.	
Evidence to recommendation synthesis	The quality of the evidence is generally weak, with some nutrigenetic associations demonstrating moderate-quality evidence for *lack* of association. Potential risks outweighed potential benefits and patients would not wish to receive dubious (scientifically invalid) nutrition information and advice. As such, the panel has made a strong recommendation *against* testing certain genetic variations.		

*Includes total cholesterol, HDL-cholesterol, LDL-cholesterol, LDL particle size, TG and/or apolipoproteins.

**Table 2:** Summary of CPG recommendations.

**Key factors**	**Resulting nutrigenetic CPG recommendations**
Main considerations of general guidelines for TG lowering through lifestyle (beyond omega-3 intake)	• Reduce intake of refined sugar/carbohydrates, alcohol and/or trans fats. • Increase physical activity.
Main considerations of general guidelines for omega-3 supplementation or dietary intake	• Do not recommend omega-3s from marine sources in patients with contraindications (e.g., fish allergy). • Monitor for potential adverse effects such as gastrointestinal discomfort or skin abnormalities. • Do not exceed 3.0 g/day EPA+DHA.
*APOE* rs429358, rs7412, EPA and/or DHA and TG	• Strong level of evidence to recommend 0.7–3.0 g/day EPA and/or DHA in E3/E4 or E4/E4 genotypes for significant TG lowering. Other genotypes are less likely to benefit from 0.7 to 3.0 g/day EPA and/or DHA for TG lowering. EPA and/or DHA may still be recommended to these patients for reasons beyond TG reduction; plasma TG levels should then be monitored accordingly. • HCPs must take location-specific regulatory requirements into consideration when deciding on EPA/DHA dosages for their patient. • Generalizable population: adult males (but not females). • Consider ethical and regulatory considerations when offering this test in a clinical setting.
31-SNP nutri-GRS, EPA+DHA and TG	• Strong level of evidence to recommend 3.0 g/day EPA+DHA for TG lowering in those who have lower nutri-GRSs. On a range of scores from −6 to +10, those with higher nutri-GRSs are less likely to benefit from 3.0 g/day EPA+DHA for TG lowering, and may exhibit increases in plasma TG levels in response. EPA+DHA should not be routinely recommended to patients with higher nutri-GRSs; these patients' plasma TG levels should be monitored accordingly if they are taking EPA+DHA supplementation for reasons beyond TG management. Individuals with nutri-GRS scores closer toward the limits of the range (e.g., −6 and +10) can be more clearly classified as responders vs. non-responders, compared to those closer to the middle of the range. • HCPs must take location-specific regulatory requirements into consideration when deciding on EPA+DHA dosages for their patient. • Generalizable population: adults with overweight/obesity.
Cases of conflicting results for *APOE* rs429358, rs7412 and 31-SNP nutri-GRS	• Given that the level of evidence is higher for the 31-SNP nutri-GRS compared to *APOE* rs429358 and rs7412, the nutri-GRS result should be used to guide practice related to TG management in lieu of the *APOE* result if results are conflicting among patients who fall within the generalizable population stated below. For example, EPA+DHA for TG reduction should be recommended to adult male patients with overweight/obesity who have a low nutri-GRS, even if they are not *APOE* E4 carriers. • Generalizable population: adult males with overweight/obesity
Other genes, SNPs and lipid/lipoprotein outcomes	• Beyond the abovementioned guidelines related to *APOE* and the 31-SNP nutri-GRS, do not provide any other nutrigenetically-guided personalized omega-3 recommendations for total cholesterol, HDL-cholesterol, LDL-cholesterol, LDL particle size, TG and/or apolipoproteins.

*Recommendation 1:* The expert panel *conditionally* recommends that in male patients (but not females), nutrigenetic testing for TG responsiveness to EPA and/or DHA omega-3s can be based on genetic testing of *APOE* SNPs (rs429358, rs7412). Adult males with the *APOE*-E3/E4 or E4/E4 genotype appear to be most likely to experience significant TG reductions in response to 0.7–3.0 g/day EPA and/or DHA; higher dosages may have greater TG lowering effects (18). It should be noted that while research suggests that up to 3.7 g/day can be effective for TG lowering, 3.0 g/day of EPA+DHA are considered pharmacological dosages by the AHA (42) and as such we have revised the upper dosage recommendation accordingly for those with the *APOE*-E3/E4 or E4/E4 genotype. Maximum EPA+DHA dosage regulations may vary depending on location therefore the HCP must take their location-specific regulations into consideration when deciding on the appropriate dose for the patient.

Given the link between these *APOE* genetic variations and Alzheimer's Disease (31), Companies/laboratories producing nutrigenetic tests that include *APOE* (rs429358 and rs7412) as it relates to plasma TG responsiveness to EPA and/or DHA, and HCPs offering such tests to patients must comply with the regulations relevant to their territory of practice, while also considering any ethical and legal implications of this test. Research laboratories testing these SNPs must work with their institutional research ethics board to determine how to proceed with *APOE* testing. How to best proceed with this test in an industry, clinical or research setting will be context specific. Consent for *APOE* genotyping should always be obtained from patients prior to completing genotyping, as they would incidentally also be learning about other disease risks, including Alzheimer's disease. Disclosure of results to patients and referral to appropriate HCPs such as genetic counselors and medical doctors for Alzheimer's disease risk counseling may be warranted if it is in accordance with the consent form signed by the patient. Thus, nutrition professionals (or company offering DTC testing) must be aware of available referral sources (e.g., medical doctors and genetic counselors) prior to offering this test. This recommendation is also conditional on any context-specific policies and regulations related to *APOE* genetic testing, as well as the patient's consent to undergo *APOE* testing and disclosure of the results related to Alzheimer's disease risk (or not).

*Recommendation 2:* The expert panel further conditionally recommends that the nutri-GRS developed by Vallée Marcotte et al. (25, 26) can be used to evaluate TG responsiveness to ~3.0 g/day of EPA+DHA. This risk score is generalizable to adults with overweight and obesity and has been studied in samples of men and women (combined) and therefore can be used for both male and female patients. The details of the nutri-GRS are outlined in Supplementary Table 2 and are described in the original studies (25, 26). EPA+DHA could be recommended to patients with a low nutri-GRS for TG reduction, however EPA+DHA should not be recommended to individuals with higher nutri-GRSs to reduce plasma TG levels. While the research conducted to date intervened with a dosage of ~3.0 g/day EPA+DHA, dosage regulations differ by location so the HCP should of course only recommend a dosage level and type that is in accordance with their location-specific regulations. Furthermore, while the precise cut-off value for classifying a low vs. high nutri-GRS has not yet been identified, cut-off values of two or lower and five or higher for low and high scores, respectively, may be used as a starting point; scores in between two and five appear to be less clear in terms of the classification as a responder or adverse responder (25). For these mid-range scores, providing nutrigenetic advice may not be appropriate at this time. In cases where EPA+DHA are recommended to adverse responders (those with high nutri-GRSs) for reasons other than plasma TG management, TG levels should be monitored.

*Recommendation 3:* Finally, the panel strongly recommends *not* to provide personalized omega-3 fatty acid recommendations for plasma lipids, lipoproteins and apolipoproteins based on genetic variation of any SNPs, outcomes and types of omega-3 beyond those specified in Recommendation 1 and 2, based on the Vallée Marcotte et al. nutri-GRS (25, 26), or *APOE* genotype given the evidence currently available. However, the committee anticipates that these current recommendations will be expanded in the future as more high-quality intervention studies are conducted.

Table 2 provides a brief overarching summary of these CPGs.

## Discussion

We have developed the first CPGs in nutrigenetics, which are specific to the genetic variability of plasma lipid/lipoprotein/apolipoprotein responses to dietary/supplemental omega-3. Two recommendations were considered conditional (level 2), related to a 31-SNP nutri-GRS and *APOE* genetic variants contributing to the TG response to omega-3 fatty acids. The recommendation being “conditional” implies variability (i.e., some would choose the intervention, some would not)—it does not imply that the evidence to support the test is weak, which is a common misconception (13). In fact, the body of evidence to support recommendations 1 and 2 is strong (5).

These CPGs can be used by HCPs and industry alike to help promote evidence-based practice in personalized nutrition. Industry should use these CPGs to inform the nutrigenetic tests and omega-3 recommendations included in their reports. The CPGs can further be used by HCPs in cases where a patient brings results from a direct-to-consumer report to their appointment; HCPs can then verify the validity of any information included in the report related to omega-3s and lipid/lipoprotein/apolipoprotein outcomes. HCPs and consumers can further cross-reference these CPGs with existing nutrigenetic tests related to omega-3s and plasma lipids/lipoproteins/apolipoproteins to determine those that are evidence-based (and those that are not), and the caveats of those that are ready for implementation into practice. Decision aids can be useful to guide clinical practice for HCPs (61), and can be particularly useful when GRADE recommendations are conditional (14). Future research should seek to develop a decision aid related to omega-3 fatty acids and TG outcomes based on genetic variation.

Moreover, the proportion of the *APOE-*E4 carriers is estimated to range from 7 to 31%, with rates variable depending on ethnicity (62). For example, Asian populations typically have lower frequencies of *APOE*-E4 carriers compared to other ethnicities (around 7%), such as individuals from Norway who have one of the highest frequencies (around 31%) (62). Based on a multi-ethnic study, Vallée Marcotte et al. found that an estimate of at least a third of the Canadian population could be responders to EPA+DHA for TG lowering according to the 31-SNP nutri-GRSs, with an even higher prevalence of responders in the European study sample (25). Notably, prevalence estimates in genetics tend to differ depending on ethnicity (62–64), so the impact of these CPGs at a population level will be variable depending on ethnicities.

There are some limitations to the present CPGs that should be noted. First, the research conducted to date for the *APOE* nutrigenetic testing described herein is generalizable to adult males only. Therefore, this test is not considered to be scientifically valid in females at this time. Similarly, the 31-SNP nutri-GRS is generalizable to adults with overweight or obesity and is therefore not yet applicable to other subsets of the population. Future research should thus include sex-stratified analyses, focus on broader target populations, and should further prioritize interventional study designs especially randomized controlled trials given that these study designs tend to lead to higher levels of evidence (13). Additionally, since the 31-SNP nutri-GRS was developed using a statistical analysis of a continuous variable, a precise cut-off value for classifying responders vs. non-responders has not yet been identified. As such, it may not be as clear for practitioners to translate the genetic results into practice recommendations for individuals in the middle of the nutri-GRS range. However, 97% of participants with nutri-GRS scores of 2 or lower responded to omega-3 for TG lowering, while 91% with nutri-GRS scores of 5 or higher were classified as non-responders in Vallée Marcotte et al. (25) and as such this may be a good starting point for clinical cut-offs. Future research should however seek to better clarify these cut-off points. The approach used here in relation to nutri-GRS and omega-3 fatty acid intake could, in the future, also be applied to the response to other nutrients and/or other health conditions.

Also, more systematic reviews are needed in the field of nutrigenetics. These could then lead to the development of other clinical practice guidelines in this field. Overall, proper nutrigenetics training for dietitians and other nutrition providers is essential. This has been further discussed in a recently developed nutrigenomics care map outlining general considerations for the integration of genetic testing into practice (65); we encourage HCPs to refer to this document to support/complement the recommendations provided in the present CPGs.

In conclusion, the results of these first CPGs in nutrigenetics should be used to guide evidence-based practice in personalized nutrition for omega-3 fatty acids and their impact on plasma lipids/lipoproteins/apolipoproteins.

## Data Availability Statement

The original contributions presented in the study are included in the article/Supplementary Material, further inquiries can be directed to the corresponding author/s.

## Author Contributions

JK was responsible for writing the first draft of the manuscript and making revisions to the guidelines. JK, VG, SD, and M-CV collaboratively developed the first draft of the guidelines. DM, JR, IR, VM, and GS revised the guideline draft. All authors approved the final guidelines.

## Funding

This project was supported through a pilot projects grant from INAF. JK was supported through postdoctoral fellowships from Canadian Institutes of Health Research (CIHR) (#430907), NUTRISS, and INAF. IR holds a Junior 2 research Scholar from the Fonds de Recherche du Québec—Santé (FRQ-S). M-CV holds a Canada Research Chair in Genomics Applied to Nutrition and Metabolic Health. None of the funders played a role in the content of these CPGs.

## Conflict of Interest

The authors declare that the research was conducted in the absence of any commercial or financial relationships that could be construed as a potential conflict of interest.

## Publisher's Note

All claims expressed in this article are solely those of the authors and do not necessarily represent those of their affiliated organizations, or those of the publisher, the editors and the reviewers. Any product that may be evaluated in this article, or claim that may be made by its manufacturer, is not guaranteed or endorsed by the publisher.

## Abbreviations

AGREE: Appraisal of Guidelines for Research and Evaluation
CPG: clinical practice guideline
DHA: docosahexaenoic acid
EPA: eicosapentaenoic acid
FDA: Food and Drug Administration
GRADE, Grading of Recommendations Assessment: Development and Evaluation
HCP: healthcare professional
nutri-GRS: nutrigenetic risk score
SNP: single nucleotide polymorphism
TG: triglycerides.
