# Supplementary material for: Clinical Practice Guidelines Using GRADE and AGREE II for the Impact of Genetic Variants on Plasma Lipid/Lipoprotein/Apolipoprotein Responsiveness to Omega-3 Fatty Acids
Source: Front Nutr. 2022 Feb 14;8:768474. doi: 10.3389/fnut.2021.768474 (PMC8883048; doi:10.3389/fnut.2021.768474)
Supplement: Supplementary file 2 [file Data_Sheet_2.PDF]

Supplementary Table 2: 31-SNP Nutri-GRS

Gene, **rs Number**	**Alleles[1]**	**Associated Points**
IQCJ-SCHIP1, rs7639707	A/G	+1
IQCJ-SCHIP1, rs62270407	C/T	-1
NXPH1, rs61569932,	G/T	+1
NXPH1, rs1990554	A/C	+1
NXPH1, rs6463808	A/G	+1
NXPH1, rs6966968	A/G	+1
NXPH1, rs28473103	A/G	-1
NXPH1, rs28673635	A/G	+1
NXPH1, rs12702829	C/T	+1
NXPH1, rs78943417	A/T	-1
NXPH1, rs293180	G/T	+1
NXPH1, rs1837523	C/T	-1
PHF17, rs1216346	C/T	+1
PHF17, rs114348423	A/G	+1
PHF17, rs75007521	G/T	-1
MYB, rs72560788	C/T	-1
MYB, rs72974149	A/G	-1
MYB, rs210962	C/T	-1
MYB, rs6933462	C/G	+1
NELL1, rs79624996	A/G	+1
NELL1, rs1850875	C/T	+1
NELL1, rs78786240	C/T	-1
NELL1, rs117114492	G/T	+1
SLIT2, rs184945470	C/T	+1
SLIT2, rs143662727	A/G	-1
SLIT2, rs10009109	C/T	+1
SLIT2, rs10009535	A/G	+1
SLIT2, rs61790364	A/G	+1
SLIT2, rs73241936	C/T	+1
SLIT2, rs16869663	A/G	+1
SLIT2, rs76015249	A/G	+1

1. Minor alleles are underlined

For individuals carrying one or two minor alleles, provide the associated number of points (either +1 or -1). For individuals homozygous for the major allele, provide 0 points. Count the overall number of points. Individuals with lower nutri-GRS are more likely to respond to approximately 3.0 g/day EPA+DHA for TG lowering.
